# Is there a need to redo many of the diagnoses of hypertension?

**DOI:** 10.1590/S1516-31802012000300007

**Published:** 2012-07-12

**Authors:** José Marcos Thalenberg, Bráulio Luna, Maria Teresa Nogueira Bombig, Yoná Afonso Francisco, Rui Manuel dos Santos Póvoa

**Affiliations:** I MD, PhD. Head of the Blood Pressure Monitoring Service, Hypertensive Cardiopathy Clinic, Universidade Federal de São Paulo (Unifesp), São Paulo, Brazil.; II MD, PhD. Full Professor of Cardiology, Hypertensive Cardiopathy Clinic, Universidade Federal de São Paulo (Unifesp), São Paulo, Brazil.; III MD, PhD. Cardiologist, Hypertensive Cardiopathy Clinic, Discipline of Cardiology, Universidade Federal de São Paulo (Unifesp), São Paulo, Brazil.; IV MD, PhD. Cardiologist and Adjunct Professor, Head of the Hypertensive Cardiopathy Clinic, Discipline of Cardiology, Universidade Federal de São Paulo (Unifesp), São Paulo, Brazil.

**Keywords:** Blood pressure determination, Hypertension, Diagnostic techniques, cardiovascular, Blood pressure monitoring, ambulatory, Headache, Determinação da pressão arterial, Hipertensão, Técnicas de diagnóstico cardiovascular, Monitorização ambulatorial da pressão arterial, Cefaléia

## Abstract

**CONTEXT AND OBJECTIVE:**

Most hypertensive subjects undergoing treatment were diagnosed solely through measurements made in the consultation office. The objective of this study was to redo the diagnosis of treated patients after new clinical measurements and ambulatory blood pressure monitoring (ABPM).

**DESIGN AND SETTING:**

Cross-sectional study conducted in an outpatient specialty clinic.

**METHODS:**

Patients with mild-to-moderate hypertension or undergoing anti-hypertensive treatment, without target organ damage or diabetes, were included. After drug withdrawal lasting 2-3 weeks, new blood pressure (BP) measurements were made during two separate visits. ABPM was performed blindly, in relation to clinical measurements. The BP thresholds used for diagnosing hypertension, white-coat hypertension, normotension and masked hypertension were: 140 (systolic) and 90 (diastolic) mmHg for office measurements and 135 (systolic) and 85 (diastolic) mmHg for mean awake ABPM (MAA).

**RESULTS:**

Evaluations were done on 101 subjects (70% women); mean age 51 ± 10 years. The clinical BP was 155 ± 18/97 ± 10 mmHg (first visit) and 150 ± 16/94 ± 11 mmHg (second visit); MAA was 137 ± 13/ 86 ± 10 mmHg. Sixty-four patients (63%) were confirmed as hypertensive, 28 (28%) as white-coat hypertensive, nine (9%) as normotensive and none as masked hypertensive. After ABPM, 37% of the presumed hypertensive patients did not fit into this category.

**CONCLUSION:**

This study showed that hypertension was overdiagnosed among hypertensive subjects undergoing treatment. New diagnostic procedures should be performed after drug withdrawal, with the aid of BP monitoring.

## RESUMO

**CONTEXTO E OBJETIVO:**

A maioria dos hipertensos em tratamento teve seu diagnóstico feito somente com medidas no consultório. O objetivo deste estudo é refazer o diagnóstico em pacientes tratados após novas medidas clínicas e monitorização ambulatorial da pressão arterial (MAPA).

**TIPO DE ESTUDO E LOCAL:**

Estudo transversal realizado em clínica de especialidade.

**MÉTODOS:**

Foram incluídos pacientes com hipertensão leve a moderada ou em tratamento anti-hipertensivo, sem lesões de órgão-alvo ou diabetes. Após suspensão do tratamento por duas a três semanas, novas medidas da pressão arterial (PA) foram feitas em duas visitas distintas. A MAPA foi feita às cegas em relação às medidas clínicas. Os limites pressóricos utilizados para o diagnóstico da hipertensão, hipertensão do avental branco, normotensão e hipertensão mascarada foram: 140 (sistólica) e 90 (diastólica) mmHg para as medidas de consultório e 135 (sistólica) e 85 (diastólica) mmHg para as médias de vigília da MAPA (MVM).

**RESULTADOS:**

Foram avaliados 101 indivíduos (70% mulheres), idade 51 ± 10 anos. PA clínica: 155 ± 18/97 ± 10 mmHg (primeira visita) e 150 ± 16/94 ± 11 mmHg (segunda visita), MVM de 137 ± 13/86 ± 10 mmHg. Sessenta e quatro (63%) pacientes foram confirmados como hipertensos, 28 (28%) como portadores de hipertensão do “avental branco”, 9 (9%) como normotensos e nenhum como tendo hipertensão mascarada. Após a MAPA, 37% de presumíveis hipertensos não se enquadravam como tal.

**CONCLUSÃO:**

Este estudo revela um excesso de diagnósticos de hipertensão em hipertensos tratados. Novos procedimentos diagnósticos devem ser realizados após suspensão da medicação, com auxílio das monitorizações da PA.

## INTRODUCTION

The diagnosis of arterial hypertension (HT) is among the most commonly seen diagnoses in clinical practice and is one of the diagnoses most subject to error. Technical problems in blood pressure (BP) measurements, low numbers of measurements made in consultation offices and the elevation of BP inherent to the medical environment (“white coat” phenomenon) are frequent error factors.^1^ The advent of BP monitoring has cast new light on the diagnosis of HT through showing the differences that may exist between measurements in the office and outside of the office, whether made dynamically (ambulatory BP monitoring, ABPM) or statically (home BP monitoring, HBPM). Thus, such differences reveal the nuances that exist between established hypertension and normotension, and have even redefined these concepts.

With regard to the new diagnostic categories created through monitoring, it has been seen that both diagnostic overestimation in the office (white coat hypertension, WCH) and underestimation (masked hypertension, MH) may have important consequences in clinical practice. Correct identification of these conditions is fundamental for avoiding unnecessary treatment (in WCH) or instituting adequate treatment (in MH).^2^ Proper attention must be given to the diagnosis of WCH in subjects with office hypertension, due to its high prevalence (15 to 30% of presumably hypertensive individuals).^3^ BP monitoring may also add prognostic value to the office measurements, with progressively higher cardiovascular risk until reaching maximum values when the three types of measurement (office, ABPM and HBPM) are high.^4^

## OBJECTIVE

Most individuals who are considered to be hypertensive are diagnosed and treated based only on office measurements. The aim of this study was to redo the diagnoses of clinically hypertensive subjects undergoing treatment after new office measurements and ambulatory blood pressure monitoring (ABPM).

## METHODS

In this cross-sectional study, 101 patients who had been referred to the Hypertensive Cardiopathy Sector of Universidade Federal de São Paulo (Unifesp) between March 2003 and March 2005 were assessed in screening consultations. The assessment consisted of a structured questionnaire and interpretation of the laboratory tests and electrocardiograms (EKG) that form part of the routine in this sector. The questionnaire included questions about: how long ago the hypertension had been diagnosed; anti-hypertensive medications used (name and dose); other medications used; status of smoking and alcohol use; other ailments present; and hospital admissions (including attendance for hypertensive crises). The inclusion criteria were that the subjects should be adults (age ³ 18 years) with a register of systolic BP (SBP) ³ 140 and < 180 mmHg or diastolic BP (DBP) ³ 90 and < 110 mmHg, or using antihypertensive medication.

Patients were excluded if they presented: SBP ³ 180 or DBP ³ 110 mmHg; a history of attendance for hypertensive crises of any origin; secondary hypertension; diabetes mellitus; atrial fibrillation; unstable or recent-onset angina; valvulopathy with functional class > 1; serum creatinine ³ 1.5 mg/dl; body mass index ³ 35; or Sokolow-Lyon index (SV1 + RV5/V6 ³ 35 mm) on EKG. Cases of moderate to severe chronic obstructive pulmonary disease were also excluded because the patients were simultaneously participating in a study that included a respiratory test.^5^ Informed consent was obtained from all patients before their inclusion in the study, and the protocol had previously been approved by the institution’s Research Ethics Committee.

The antihypertensive medication of the patients selected was withdrawn for two to three weeks.^6^ Proper care was taken to avoid cases of rebound hypertension due to the withdrawal. Patients presenting headache were given prior guidance regarding the absence of a causal relationship between primary hypertension and this symptom,^7^ and were told simply to take their usual analgesics and/or anti-migraine drugs for the duration of the suspension of the antihypertensive medication. A contact telephone number was provided in case any patients had queries during the suspension period.

When the patients came back to the consultation office, BP was determined using a mercury sphygmomanometer (Wan Med, São Paulo, Brazil) graduated every 2 mm, with cuffs of adequate size for the upper arm circumference. BP was measured in both arms with the patient seated, and the arm with the higher BP was chosen. After waiting for two minutes, another measurement was made in the same arm. If the DBP differed by more than 5 mmHg, the procedure was repeated until measurements with a smaller difference were obtained.

The patient was then immediately fitted with a monitor in order to perform 24-hour ABPM (Dyna-MAPA, Cardios Sistemas, São Paulo, Brazil; equivalent to Mobil-O-Graph, I.E.M., Stolberg, Germany), with an appropriate cuff placed on the non-dominant arm. The device was programmed to measure BP every 15 minutes while the patient was awake and every 30 minutes while asleep. The times of going to sleep and waking up were established individually and checked using diary records.

On the next day, after the monitor had been removed, the same procedures as described above were performed to measure BP at a new consultation. The ABPM results were interpreted in a blinded manner in relation to the clinical measurements, and were interpreted in accordance with the Brazilian guidelines.^8^ We considered that records consisting of at least 50 BP measurements taken while the patient was awake were valid.

The clinical measurements were made by a trained physician, who performed them without any observer admitted during the procedure, in order to avoid raising the patient’s BP.^9^ All the clinical measurements were obtained in the mornings, between 11:00 a.m. and 12:00 noon.

The following diagnostic criteria were used: Hypertension (HT), if the clinical BP was ³ 140 (systolic) or 90 (diastolic) mmHg at both medication-free consultations (also in the screening, if the patient had not been under medication) and the mean ABPM while awake was ³ 135 (systolic) or 85 (diastolic) mmHg; white coat hypertension (WCH) if the clinical measurements were as above, and the mean ABPM while awake was < 135/85 mmHg; masked hypertension (MH) if the clinical BP was < 140/90 mmHg at both consultations or at the second consultation, and the mean ABPM while awake was ³ 135 (systolic) or 85 (diastolic) mmHg; normotension (NT) if the clinical BP was as above and the mean ABPM while awake was < 135/85 mmHg.

Considering a mean prevalence of 20% WCH, alpha error of 5% and statistical power of the sample of 90%, we estimated that it would be necessary to have 92 individuals with a presumed diagnosis of sustained hypertension. The results were expressed as means and standard deviations (SD). Proportions were expressed as percentages. We used the Microsoft Excel software to calculate the means and SD.

## RESULTS

The participants in this study comprised 71 women and 30 men, with a mean age of 50.7 ± 10 years. Of these, 57 patients were white, 40 were black and four were East Asian. Ten patients (seven women) were over the age of 65 years. Ninety-four patients were regularly using antihypertensive medication: 17 using monotherapy, 74 using two drugs and three using three drugs (these last three at sub-optimal doses). The drugs used belonged to the following categories: thiazide diuretics, beta-blockers, dihydropyridine calcium channel blockers and angiotensin-converting enzyme inhibitors. All of these were available through the public healthcare system of the city of São Paulo.

The mean BP measurements in the consultation office were: 155 ± 18/97 ± 10 mmHg at the first visit and 150 ± 16/94 ± 11 mmHg at the second visit. The mean ABPM while awake was 137 ± 13/86 ± 10 mmHg.

**Figure 1.**
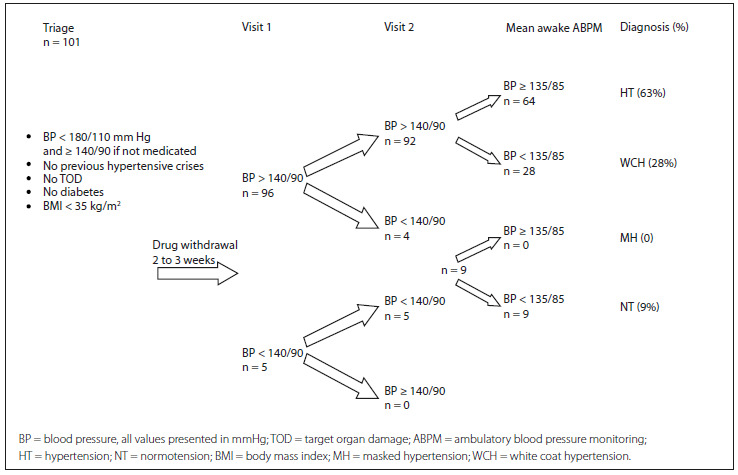
Diagnostic algorithm and results.

Nine patients (9%) presented normal clinical BP and ABPM measurements, even after withdrawal of the medication. Sixty-four patients (63%) were classified as HT and 28 (28%) as WCH. None of the patients was classified as MH. Among the seven patients who were not using medications at the time of the screening, five of them were classified as HT after ABPM and two, as WCH. After the ABPM, 37% of the supposed hypertensive patients were found not to fit into this category. Figure 1 shows the diagnostic algorithm for the study and its results. Table 1 shows the mean BP according to diagnostic category.

**Table 1.** Mean blood pressure (BP) in mmHg according to diagnostic category

	Visit 1	Visit 2	Mean awake ambulatory blood pressure monitoring
Hypertension	158 ? 18/101 ? 10	154 ? 16/99 ? 10	144 ? 10/91 ? 8
White coat hypertension	150 ? 13/92 ? 8	146 ? 11/89 ? 9	125 ? 6/77 ? 6
Normotension	141 ? 20/87 ? 10	130 ? 8/81 ? 7	124 ? 6/74 ? 5

The telephone number that was made available for contacts in the event of queries during the medication suspension period was only used by two female patients, who both presented chronic headache. They presented pain episodes that were similar to those that had been experienced while under antihypertensive treatment. They were advised again to only take the analgesics that they were already using regularly, and they came back to the office without intercurrences, to continue with the protocol. A third female patient went through a similar situation, did not get in touch and dropped out of the study.

## DISCUSSION

In this study, hypertensive individuals’ diagnoses were redone after their medication had been withdrawn, with new measurements at two consultations in the office and through ABPM. At the end of this procedure, 37% of these individuals were found not to fit into the initial diagnostic category. This result suggests that hypertension is being diagnosed excessively, with possible clinical and economic consequences for the population and for the healthcare system.

Variations in blood pressure are a challenge with regard to clinical diagnosis and hypertension control. The spontaneous fall in BP between the first and second consultations (Table 1) was probably due to attenuation of the patients’ alert reaction to the medical environment. This attenuation occurs through repeated visits (habituation effect). This confirms that there is a need to have at least two consultations after screening, in order to make clinical diagnoses of hypertension.^10^^,^^11^ Through this procedure, we found that nine patients presented normal BP (9% of the initial sample): all of them had previously been taking antihypertensive medication without showing any symptoms suggestive of hypotension.

WCH seems to be a conditioned response to the medical environment that does not disappear with time.^12^ There is no need for antihypertensive medication, except in cases of elevated cardiovascular risk. It is possibly a pre-hypertensive state,^13^^,^^14^ which therefore requires control more frequently than for normotensive individuals. For WCH to be diagnosed, monitoring outside of the office is essential. In our study, 28% of the sample was shown to have this condition after ABPM. This result corroborates the affirmation of the European Hypertension Society: “In truth, it must be admitted that it is difficult to escape the conclusion that all patients in whom a diagnosis of hypertension is being contemplated based on office/clinic BP, should have ABPM to exclude WCH”.^15^ Home BP monitoring (HBPM) may also be used for this same purpose.^16^

We did not detect any cases of masked hypertension. This is a condition that is generally investigated in supposedly normotensive individuals who present target organ damage. Our result was not surprising, given that we were only analyzing individuals who were supposedly hypertensive and did not have target organ damage, in accordance with the exclusion criteria adopted. Masked hypertension carries a cardiovascular risk that is close to that of sustained hypertension, and should be treated in the same way.^17^

Withdrawal of the antihypertensive medication is the fundamental measure for redoing the diagnosis, since in such cases, the medication treatment acts as a confounding factor. This is an ethical and safe procedure among hypertensive individuals without complications, provided that the due precautions are taken in relation to drugs that, if withdrawn, may cause rebound hypertension, such as clonidine.^18^ All of the patients accepted the proposed withdrawal well, after being given proper explanations. With regard to the three patients who were using regimens of three medications belonging to different classes, none of them fitted into the category of resistant hypertension according to the criterion of the doses used (for example, patient no. 55 was using the following daily doses: hydrochlorothiazide 25 mg, captopril 50 mg and propranolol 40 mg). In our study, all the patients had their medication withdrawn only once. As expected, no cases of rebound hypertension occurred during the suspension period.

The greater number of women in the sample reflects the frequencies of users of the Hypertensive Cardiopathy Sector (women to men, 7:3). This was not a surprise to us, since in Brazil, women seek healthcare services more than men do.^19^ The diagnosis of WCH is of great importance among women, given that they are more susceptible to this condition.^20^ WCH was found in 35% of the women and 20% of the men in our sample, after excluding the nine normotensive patients.

There is often an association between headache and hypertension,^21^ since these are both highly prevalent conditions worldwide. Nonetheless, several studied using ABPM and/or clinical measurements have suggested that there is no causal relationship between these two conditions.^22^^,^^23^^,^^24^^,^^25^^,^^26^^,^^27^ In a recent prospective study on a large population, it was even found that there was an inverse relationship between headache and blood pressure levels, and it was suggested that this was related to the phenomenon of hypertension-associated hypalgesia.^28^^,^^29^ Primary hypertension is only a proven cause of headache in cases of hypertensive encephalopathy, a hypertensive emergency in which cerebral edema is associated. The myth of hypertension as a cause of headache may contribute towards lowering the adherence to antihypertensive treatment, if absence of pain is taken by patients to be an indicator of controlled hypertension. In the present study, this clarification was provided to the patients with headache and was an important part of the screening consultation.

Our study found that 9% of the patients were normotensive, which was an unexpected find among hypertensive patients undergoing treatment at a reference center for hypertension. This suggests that the initial clinical diagnosis had not been made with the due care, such that the patient was referred to the specialized service already under medication and was accepted by this service as hypertensive. In our sample, after withdrawal of the medication, four of these patients were found to have normal BP at the second consultation and five of them, at both consultations. If these individuals had not had their diagnoses redone, they would have been considered to be hypertensive patients and would have continued to undergo treatment, if only because they did not present any symptoms of hypotension when under medication.

Correctly diagnosed hypertension is an absolute necessity for any healthcare system. The financial cost of unduly treating cases of WCH may be high. In a study conducted on 255 hypertensive individuals, it was seen after ABPM that 21% had WCH. If only the true hypertensive individuals had been treated, this would have resulted in savings that this study estimated to be US$ 110,000 over a six-year period.^30^ In another study with a sample of 62 hypertensive patients, of whom 26% were found to present WCH after ABPM, the financial cost of using this method started to be recompensed from the third year of use onwards.^31^ This is something to be taken into account in relation to a highly prevalent disease for which the treatment is lifelong. So far, there have not been any studies evaluating the costs of the possible iatrogenic hypotensive effects induced by improper or excessive use of antihypertensive medication, such as falls and episodes of myocardial ischemia in coronary disease patients.

## CONCLUSIONS

In conclusion, the present study detected a high prevalence of non-hypertensive individuals among presumed clinically hypertensive outpatients undergoing treatment, who were all non-diabetic and did not present target organ damage. This finding indicates that there is a need to redo the diagnosis among hypertensive individuals with this profile, with the aid of out-of office BP monitoring.

This study also suggests that, in relation to the inclusion criteria for clinical trials, individuals whose diagnosis of hypertension was made only with office measurements should not be taken *a priori* to be true hypertensive subjects, just because they are using antihypertensive medication.
